# Embryonic and mature astrocytes exert different effects on neuronal growth in rat ventral mesencephalic slice cultures

**DOI:** 10.1186/s40064-015-1362-3

**Published:** 2015-09-28

**Authors:** Sanaz Hashemian, Caitriona O’Rourke, James B. Phillips, Ingrid Strömberg, Sara af Bjerkén

**Affiliations:** Department of Integrative Medical Biology, Umeå University, Umeå, Sweden; Department of Life Health and Chemical Sciences, The Open University, Walton Hall, Milton Keynes, MK7 6AA UK; Biomaterials and Tissue Engineering, UCL Eastman Dental Institute, 256 Gray’s Inn Road, London, WC1X 8LD UK

**Keywords:** Organotypic culture, Ventral mesencephalon, Mature astrocytes, Developmental stages

## Abstract

One obstacle with grafting of dopamine neurons in Parkinson’s disease is the insufficient ability of the transplant to reinnervate the host striatum. Another issue is the prospective interaction between the donor fetal tissue and the adult astrocytes of the host. To study nerve fiber growth and its interaction with immature/mature astrocytes, ventral mesencephalic (VM) organotypic rat tissue cultures from embryonic days (E) 12, E14, and E18 were studied up to 35 days in vitro (DIV), and co-cultures of E14 VM tissue and mature green fluorescent protein (GFP)-positive astrocytes were performed. Generally, nerve fibers grew from the tissue slice either in association with a monolayer of migrated astroglia surrounding the tissue (glial-associated), or distal to the astroglia as non-glial-associated outgrowth. The tyrosine hydroxylase (TH)-positive glial-associated nerve fiber outgrowth reached a plateau at 21 DIV in E12 and E14 cultures. In E18 cultures, TH-positive neurons displayed short processes and migrated onto the astrocytes. While the non-glial-associated nerve fiber outgrowth dominated the E14 cultures, it was found absent in E18 cultures. The GFP-positive cells in the VM and GFP-positive astrocyte co-cultures were generally located distal to the monolayer of migrated fetal astrocytes, a few GFP-positive cells were however observed within the astrocytic monolayer. In those cases TH-positive neurons migrated towards the GFP-positive cells. Both the non-glial- and glial-associated nerve fibers grew onto the GFP-positive cells. Taken together, the glial-associated growth has limited outgrowth compared to the non-glial-associated nerve fibers, while none of the outgrowth types were hampered by the mature astrocytes.

## Background

One treatment strategy for reducing the symptoms in Parkinson’s disease is to transplant fetal dopamine producing tissue into the dopamine-depleted striatum of parkinsonian patients, restoring the dopamine input to the striatum. Preclinical studies demonstrate compensation of motor deficits and improvements such as functional synaptic connections (Björklund and Stenevi 1979; Perlow et al. 1979; Mahalik et al. 1985; Bolam et al. 1987; Clarke et al. 1988; Strömberg et al. 1988), normalized striatal firing rate (Strömberg et al. 1985; Fisher et al. 1991), and long-term graft survival (Freed et al. 1980; Strömberg and Bickford 1996). One of the main problems with grafting is the insufficient reinnervation of the striatum (Barker et al. 1996), a technique using several implantation sites to cover the dopamine depleted-striatum was therefore developed to cover a larger volume of the dopamine-depleted brain (Nikkhah et al. 1994).

Knowledge about the interplay between nerve fibers and surrounding cells, such as glia, is important to understand the mechanism behind the reinnervational arrest of the graft. In vitro techniques, such as organotypic tissue culture, are useful to monitor the interaction between dopaminergic neurons and astrocytes. In organotypic tissue cultures of fetal ventral mesencephalon (VM), two morphologically and temporally different types of dopaminergic nerve fibers are observed (Johansson and Strömberg 2002). The first wave of nerve fibers that is formed is independent of the presence of astroglia, and is called the non-glial-associated nerve fiber outgrowth, and is not persistent over time (Berglöf et al. 2007). The second wave, which appears around 7 days in vitro (DIV), is dependent on the presence of astrocytes and is called glial-associated nerve fibers. These nerve fibers are persistent over time, and always grow onto astrocytes. The two different nerve fiber growth patterns are present in/and reported from cultured tissue plated at embryonic day 14 (E14) in rats. Tissue obtained from later developmental ages, e.g. E18, reveal another pattern of interaction between nerve fibers and astrocytes, with migrating neurons instead of nerve fiber formation and total absence of non-glial-associated nerve fiber outgrowth (af Bjerkén et al. 2008; Hashemian et al. 2014). However, long-term monitoring of the interaction between the nerve fibers and astrocytes is yet to be performed, and may generate valuable information about the reinnervational process and bring understanding to why the graft reinnervation of the host is terminated.

The results achieved from the previous tissue culture studies indicate that neuronal growth is depending on the age of the astrocytes. Adult astrocytes, regardless of their anatomical location, occupy a large domain in the brain, which is composed of a large number of fine processes, termed ‘peripheral astrocyte processes’ (PAPs) (Derouiche and Frotscher 2001; Bushong et al. 2002). PAPs extensively contact synapses and are considered primary sites for active astrocyte and neuronal signaling (Reichenbach 2009). While astrocytes are largely generated during the first postnatal week (Bandeira et al. 2009; Ge et al. 2012), PAPs are not induced until several weeks later (P14 to P26) (Bushong et al. 2004; Freeman 2010). The present study focuses on interaction between dopamine nerve fibers and astrocytes of different embryonic ages to further elucidate the interplay between astrocytes and neuronal growth at long-term time points. The different embryonic ages were chosen based on the peak ontogeny of dopaminergic neurons, occurring between E12-15 (Hanaway et al. 1971; Missale et al. 1998; Gates et al. 2006), where TH expression begins around E12 (Specht et al. 1981; Foster et al. 1988), and around E14, dopamine can be visualized (Olson and Seiger 1972; Voorn et al. 1988). The peak of astrogliogenesis occurs late prenatal to early postnatal stages, that is E18 to postnatal day (P) 7 (Sauvageot and Stiles 2002; Tien et al. 2012), and therefore E18 was chosen. The use of organotypic tissue cultures of VM, prepared from different embryonic ages, allows for long-term monitoring of the nerve fiber-glia interactions. In addition, to the long-term VM cultures of different developmental stages, co-cultures of E14 VM tissue and mature green fluorescent protein (GFP)-positive astrocytes were performed to observe the effects of mature astrocytes on nerve fiber growth from embryonic tissue pieces.

## Results

### Astrocytic migration and nerve fiber outgrowth in E12 VM culture

**Fig. 1:**
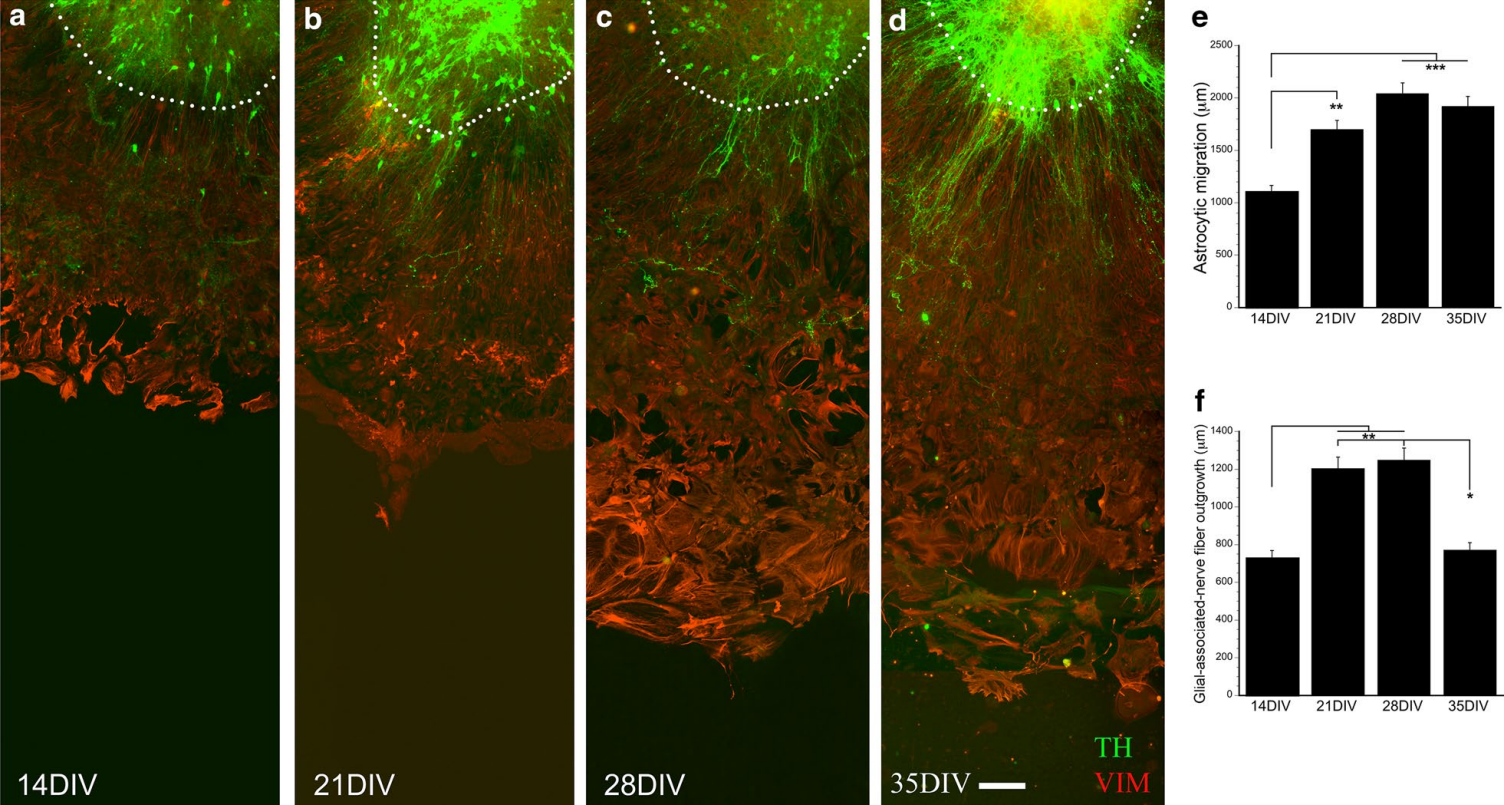
Astrocytic migration and nerve fiber outgrowth in E12 VM cultures at 14–35 DIV. E12 VM cultures were fixed at 14 DIV (**a**), 21 DIV (**b**), 28 DIV (**c**), and 35 DIV (**d**). Vimentin-positive (VIM) astrocytes migrated and formed a monolayer surrounding the tissue piece, and reached significantly longer distances at 21 DIV compared to 14 DIV (**e**). The distances that the glial-associated nerve fiber outgrowth had reached was significantly increased at 21 DIV and 28 DIV compared to 14 DIV, however at 35 DIV this type of outgrowth had a significant decrease comparing to 21 DIV and 28 DIV cultures (**f**). The non-glial-associated nerve fibers were absent at all the time points evaluated. TH = Alexa 488, vimentin = Alexa 594, 14 DIV n = 12, 21 DIV n = 11, 28 DIV n = 11, 35 DIV n = 8, *p < 0.05, **p < 0.01, ***p < 0.001. *Scale bar* **a**–**d** = 100 µm

Vimentin-positive astrocytic migration from the tissue slice in E12 VM cultures reached significantly longer distances at 21 DIV compared to 14 DIV, and had reached a plateau at 21 DIV (*F*_3,41_ = 11.723, *p* < 0.001, one-way ANOVA; Fig. 1a–e). TH-positive glial-associated nerve fiber outgrowth was longer at 21 DIV and 28 DIV compared to 14 DIV. However, at 35 DIV the outgrowth was retracted and was significantly shorter compared to that measured at 21 and 28 DIV (*F*_3,41_ = 7.688, *p* < 0.001, one-way ANOVA; Fig. 1f). The non-glial-associated nerve fiber outgrowth was in general poor (Fig. 1a–d). Thus, the glial-associated TH-positive nerve fiber outgrowth was reduced at the latest time point and poor non-glial-associated growth was present at any time point investigated.

### Astrocytic migration and nerve fiber outgrowth in E14 VM culture

**Fig. 2:**
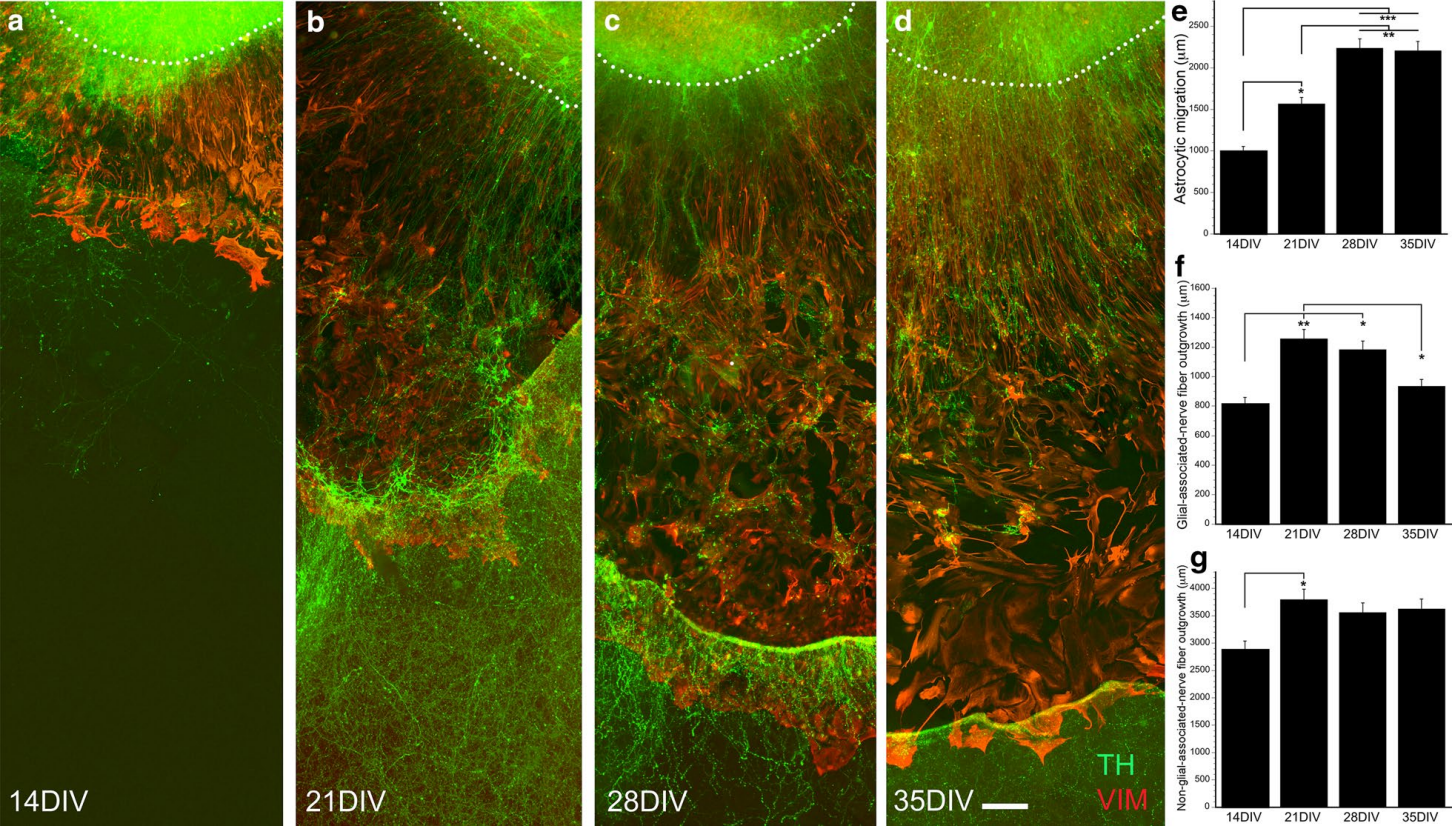
Astrocytic migration and nerve fiber outgrowth in E14 cultures at different time points. E14 VM cultures were fixed at 14 DIV (**a**), 21 DIV (**b**), 28 DIV (**c**), and 35 DIV (**d**). Vimentin-positive (VIM) astrocytes migrated significantly longer distances at 21 DIV compared to 14 DIV, and the distance was further strengthened at 28 DIV and 35 DIV (**e**). The glial-associated nerve fiber outgrowth was significantly increased at 21 DIV compared to 14 DIV, however, at 35 DIV it was shorter compared to 21 DIV cultures (**f**). The non-glial-associated nerve fiber outgrowth reached to longer distances at 21 DIV compared to 14 DIV (**g**). TH = Alexa 488, vimentin = Alexa 594, 14 DIV n = 10, 21 DIV n = 17, 28 DIV n = 11, 35 DIV n = 11, *p < 0.05, **p < 0.01, ***p < 0.001. *Scale bar* **a**–**d** = 100 µm

Astrocytes migrated for longer distances from the tissue piece at 21 DIV compared to 14 DIV, and at 28 and 35 DIV the difference was significantly strengthened (*F*_3,48_ = 15.902, *p* < 0.001, one-way ANOVA; Fig. 2a–e). The glial-associated nerve fiber outgrowth was significantly enhanced at 21 and 28 DIV compared to 14 DIV, while it was decreased at 35 compared to 21 DIV (*F*_3,48_ = 6.743, *p* < 0.001, one-way ANOVA; Fig. 2f). The non-glial-associated nerve fibers had increased in length at 21 compared to 14 DIV (*F*_3,45_ = 3.104, *p* < 0.05, one-way ANOVA; Fig. 2g). At later time points in vitro, these nerve fibers displayed a dotted appearance, although some intact fibers were observed, and reached approximately three times the length of the glial-associated nerve fibers. Thus, neither the migration of astrocytes nor the growth of glial-associated TH-positive nerve fibers was different from results found in E12 cultures, however, in E14 cultures the non-glial-associated nerve fibers were present.

### Astrocytic migration and nerve fiber outgrowth in E18 VM culture

**Fig. 3:**
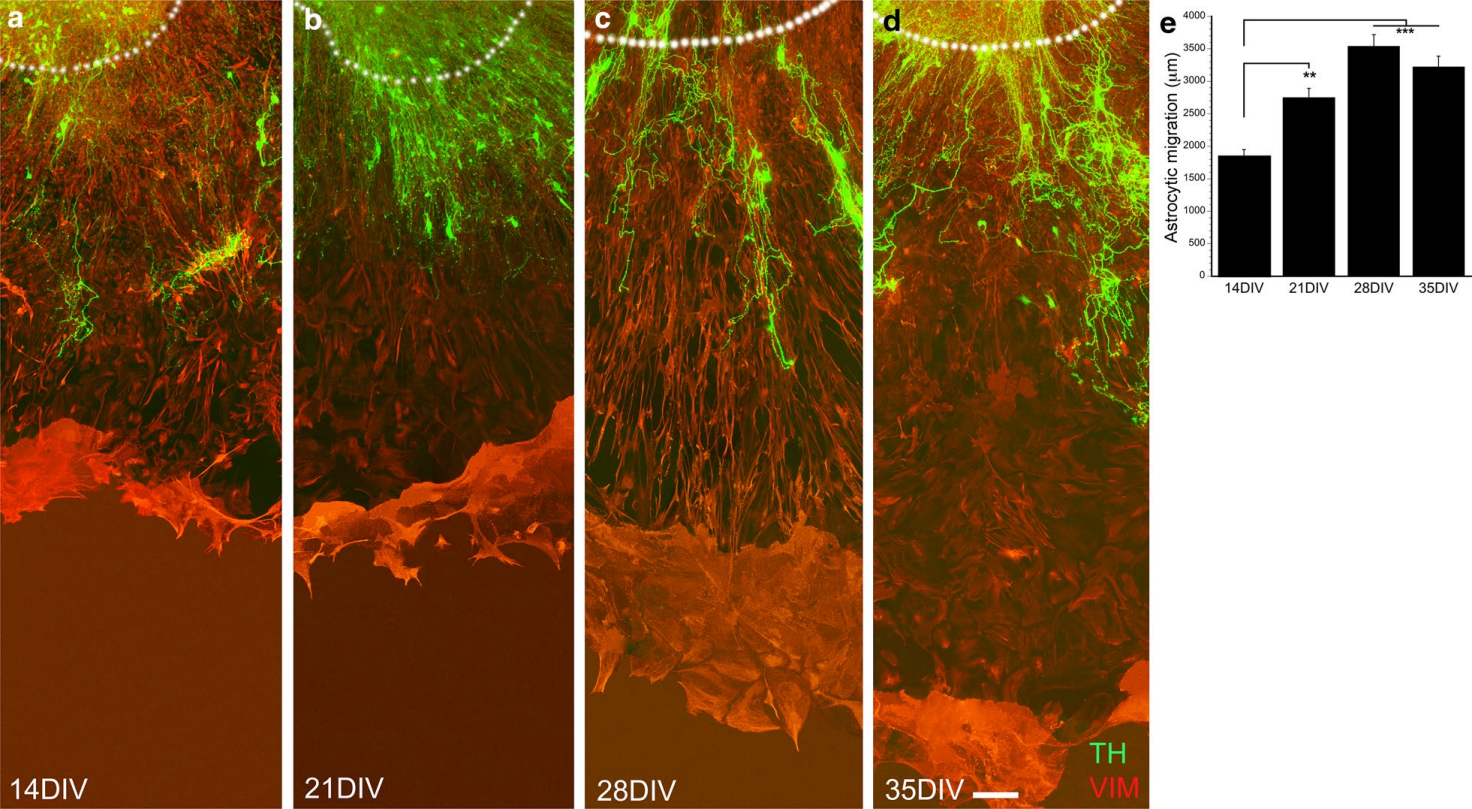
Astrocytic and neuronal migrations in E18 cultures at different time points. E18 VM cultures were evaluated at 14 DIV (**a**), 21 DIV (**b**), 28 DIV (**c**), and 35 DIV (**d**). Vimentin-positive (VIM) astrocytes reached to significantly longer distances at 21 DIV, 28 DIV and 35 DIV compared to 14 DIV cultures (**e**). TH-positive neuronal migration was seen at all time points (**a**–**d**). *White dots* mark the circumference of the tissue piece. TH = Alexa 488, vimentin = Alexa 594, 14 DIV n = 17, 21 DIV n = 19, 28 DIV n = 8, 35 DIV n = 9, **p < 0.01, ***p < 0.001. *Scale bar* **a**–**d** = 100 µm

Astrocytic migration from the tissue slice was enhanced at 21 compared to 14 DIV and reached significantly longer distances at 28 and 35 DIV (*F*_3,52_ = 13.342, *p* < 0.001, one-way ANOVA; Fig. 3e). The characteristics of E18 cultures were neural migration, which was seen at all times points in vitro (Fig. 3a–d). Furthermore, the non-glial associated nerve fibers were absent. The glial-associated nerve fibers were, however, present, but due to migration of neurons and thereby difficulties to define the boarder of the tissue culture, they were not quantified.

### Co-culture of VM tissue and GFP-positive cells

**Fig. 4:**
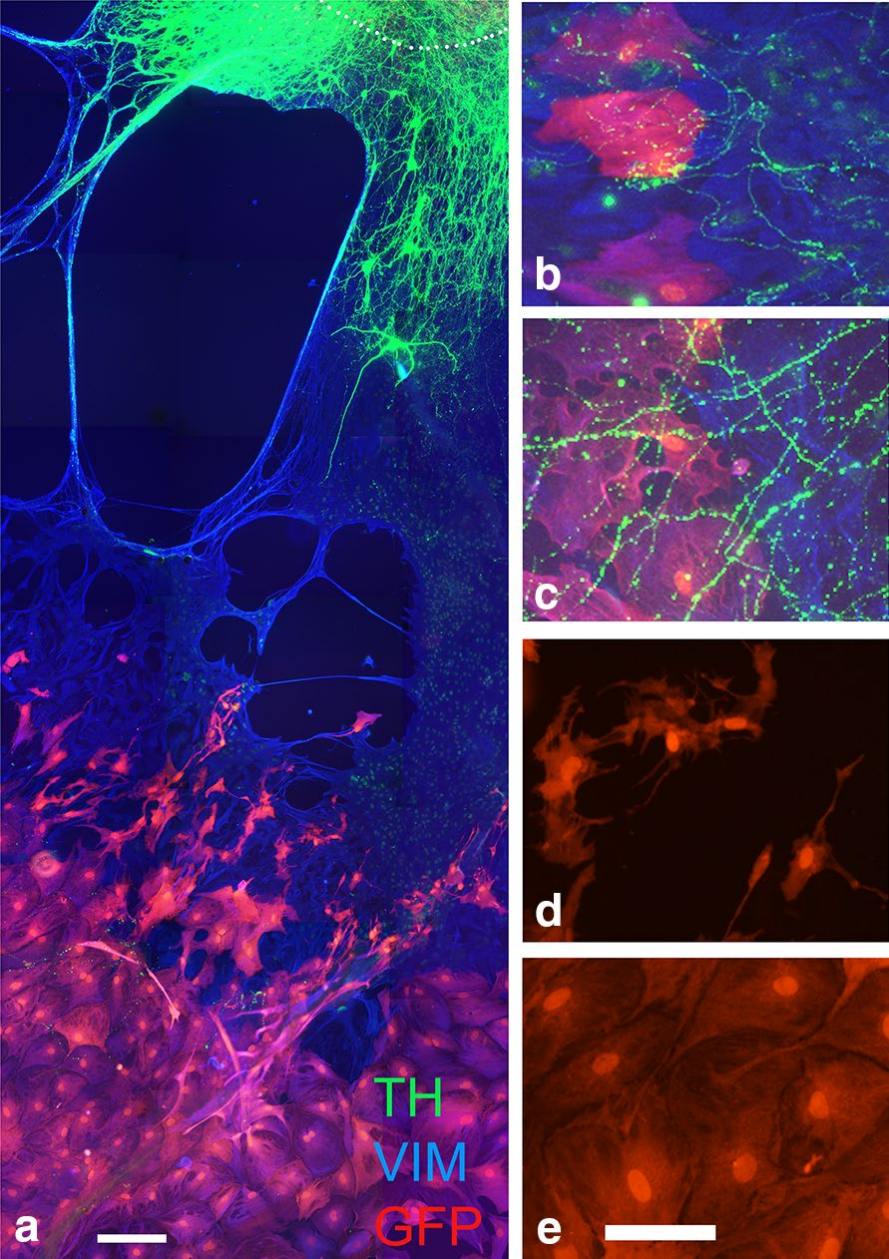
Co-culture of E14 VM and GFP-positive adult astrocytes. E14 VM-GFP-positive astrocytic co-cultures were evaluated at 21 DIV (**a**–**e**). Neurons migrated on the vimentin-positive (VIM) astrocytic network originating from the VM tissue piece (**a**). Both the glial-associated nerve fibers (**b**) and non-glial-associated nerve fibers (**c**) grew in the presence of GFP-positive cells. GFP-positive cells had different morphology depending on their distance to the tissue piece, i.e. if they were closer to the tissue piece they were smaller with small processes (**d**), and when they were located further away from the tissue piece, they had polygonal shapes (**e**). TH = *green*, vimentin = *blue*, GFP-positive = *red*. *Scale bars* **a** = 100 µm, **b**–**e** = 50 µm

Since both the non-glial- and glial-associated nerve fibers were present in E14 cultures, and both types reached a maximal outgrowth at 21 DIV, co-cultures of VM and GFP-positive mature astrocytes were studied in E14 tissue at 21 DIV. The vimentin-positive/GFP-negative astrocytes, derived from the VM tissue piece, had migrated from the tissue slice and formed a monolayer as seen in the single cultures described above. However, their organization was changed near the tissue slice from being a confluent monolayer as seen in single slice cultures to instead form a network of long vimentin-positive processes, leaving large areas empty between the processes (Fig. 4a). At longer distances, vimentin-positive/GFP-negative astrocytes were large and polygonal and formed a confluent monolayer. GFP-positive cells were organized in a monolayer distal to the GFP-negative astrocytes that had migrated from the tissue piece, however some single GFP-positive astrocytes were found closer to the VM tissue slice. The morphology of the GFP-positive astrocytes were large and polygonal, when located distal to the astrocytes derived from the tissue piece, while smaller in size and with thin processes when placed in the near vicinity of astrocytes derived from the tissue piece (Fig. 4d, e). TH-positive neurons had migrated from the tissue slice as seen in the E18 cultures (Fig. 4a), however both glial-associated and non-glial-associated growths were found. The glial-associated TH-positive nerve fibers followed the thin, long vimentin-positive fibers, and grew over the GFP-positive astrocytes (Fig. 4b). In addition, also non-glial-associated nerve fibers were found in coexistence with GFP-positive cells. Thus, the presence of the adult GFP-positive cells had no inhibitory effect on TH-positive nerve fiber outgrowth, since both glial-associated- and non-glial-associated nerve fibers were observed growing on the GFP-positive cells (Fig. 4b, c).

## Discussion

The results from the present study revealed that the outgrowth from TH-positive neurons at long-term time points in vitro was most prominent in E14 cultures, where TH-positive nerve fibers were present both on the monolayer of migrated astrocytes and beyond this astrocytic monolayer, while TH-positive outgrowth in E12 and E18 tissue cultures was restricted to the migrating astrocytes. In E18 tissue cultures, the TH-positive outgrowth was poor, and TH-positive neurons had migrated onto the astrocytic monolayer. The astrocytes migrated away from the tissue slice and formed a confluent monolayer that reached a maximal distance from the tissue slice after 21 DIV. The astrocytic distribution was similar for E12, E14, and E18 cultures. The growth from TH-positive nerve fibers was not hampered by the presence of mature GFP-positive astrocytes.

The two types of nerve fiber outgrowths, depending on the absence or presence of astrocytes, seen in VM tissue cultures, is a general phenomenon also observed in tissue cultures from other CNS areas (Hashemian et al. 2014). From earlier studies it was suggested that the non-glial-associated growth was continuously growing, as long as the astrocytes did not migrate (Johansson and Strömberg 2003; Berglöf et al. 2007; af Bjerkén et al. 2008), while in the present study these nerve fibers appeared to reach a plateau at 21 DIV. The differences in the results might be because the non-glial-associated growth has not been followed for such a long time in culture before. Their presence was dominant in E14 compared to E12 and E18 cultures, which means that they are produced within a narrow time window. Alternatively, the non-glial-associated nerve fibers were generated but did not survive up to 14 DIV. The glial-associated nerve fiber formation, on the other hand, was generated in both E12, E14, as well as in E18 cultures. The growth limit for these nerve fibers was also reached at 21 DIV, and even though the astrocytes continued to migrate for longer distances than the nerve fibers reached, nerve growth had been terminated. Thus, the early termination of reinnervation seen after grafting of fetal VM tissue into the dopamine-depleted striatum appears as a general phenomenon correlating with the time schedule for nerve fiber growth rather than presence or absence of astrocytes, this since similar limited nerve fiber outgrowth was found also in the cultures. This theory is further supported by studies demonstrating that, when grafted to the rat striatum, human nerve fibers are able to innervate for longer distances compared to rat nerve fibers (Strömberg et al. 1992; Wictorin et al. 1992; Strömberg and Bickford 1996).

In E18 cultures, nerve fiber growth was present, although hard to measure since neurons frequently had migrated onto the monolayer of astrocytes. During development, VM dopamine neurons migrate along radial glia and then tangentially to their final destination guided by already formed axons (Shults et al. 1990; Kawano et al. 1995). Migration of the dopamine neurons is mediated by stromal cell-derived factor 1 (CXCL12) via its receptor CXCR4 and reelin via the ApoER2 and VLDL receptors in the radial and tangential directions, respectively (Bushong et al. 2002; Sharaf et al. 2013; Yang et al. 2013). The migration is terminated at E18 (Shults et al. 1990; Sharaf et al. 2013), which was also one of the chosen time points for tissue harvesting/starting of primary cell cultures, and where neuronal migration was found. This migratory behavior among the TH-positive cells in E18 tissue cultures might be explained by the presence of GABA neurons (Berglöf et al. 2007). This since, while the generation of dopamine neurons is terminated at E18, the GABA neurons are still generated and their migration is modulated by already existing dopamine neurons (Vasudevan et al. 2012). Thus, the dopamine neurons might stay in their position while the GABA neurons migrate, resulting in changed location of the dopamine neurons in relation to other neurons. This may explain why the TH-positive neurons mostly are located to the periphery of the tissue slice at longer time points in vitro, which also is a common finding in VM grafts. This finding may, however, not explain why the neurons follow the migrating astrocytes. It has been demonstrated though that inhibition of astrocytic production of chondroitin sulfate proteoglycans (CSPGs) hampers neuronal migration (Hashemian et al. 2014), indicating that CSPGs, produced by astrocytes older than E14, are involved in the neuronal migration seen in E18 cultures. This was confirmed by the results from the co-cultures where neuronal migration was promoted by the presence of GFP-positive mature astrocytes.

The addition of mature astrocytes to the VM cultures was performed to understand the interaction between dopamine nerve fiber growth and mature astrocytes. The GFP-positive astrocytes are besides vimentin-positive also glial fibrillary acidic protein (GFAP)-positive, indicating that they are mature and more reactive than those derived from the tissue slice (East et al. 2010). Although all the astrocytes in the E14 cultures, in this case co-cultured with mature GFP-positive astrocytes, demonstrate immunoreactivity towards vimentin, there are some GFAP-positive astrocytes present in pure E14 cultures as well (Johansson and Strömberg 2002). These astrocytes are found close to the border of the tissue slice and display long processes radiating from the tissue piece, and does not appear morphologically rounded as the astrocytes that migrate from the tissue slice. Interestingly, while dopamine nerve fibers pass the GFAP-positive area in the E14 tissue slice, the TH-positive nerve fibers in the cultures were neither attracted to the mature GFP-positive astrocytes nor repelled by them. Thus, the hampered regeneration after for instance spinal cord injury due to the presence of GFAP-positive astroglia does not appear to be crucial for regeneration of TH-positive nerve fibers.

In conclusion, there is a critical time window for achieving long-distance nerve fiber outgrowth instead of neuronal migration. The presence of mature astrocytes appears not to inhibit these long-distance growing nerve fibers, a finding which might be important for future grafting studies.

## Methods

### Animals

Organotypic tissue cultures were performed using fetal ventral mesencephalon (VM) obtained from Sprague–Dawley rat embryos. Animals were kept in a 12:12 h light–dark cycle with ad libitum access to food and water. All experiments were approved by the local ethics committee, and animals were handled in accordance with the European Communities Council Directive. Primary astrocyte cultures were obtained from genetically modified neonatal Sprague–Dawley rats (β-actin-eGFP reporter line) in accordance with the UK Animals (Scientific Procedures) Act (1986) and the Open University animal ethics advisory group.

### Single ventral mesencephalic cultures

VM of fetuses from E12 (number of experiments = 5), E14 (number of experiments = 7), and E18 (number of experiments = 6) were dissected under sterile conditions using a dissection microscope, and chopped into 300 μm coronal slices. The slices were transferred to Dulbecco’s modified Eagle’s medium (DMEM; Gibco, Stockholm, Sweden) and cut in the midline, each tissue piece giving rise to one culture. The tissue pieces were plated in a mixture of chicken plasma (Sigma-Aldrich, Stockholm, Sweden) and thrombin (1000 U/ml; Sigma-Aldrich, Stockholm, Sweden) on poly-d-lysine (PDL; 5 mg/100 ml dH_2_O; Sigma-Aldrich, Stockholm, Sweden) -coated coverslips (12 × 24 mm). After coagulation of the plasma/thrombin clot, the coverslips were placed in a 15 ml Falcon tube containing 0.9 ml medium. The tubes were then inserted into a rotating “roller-drum” (0.5 rpm) in an incubator at 37 °C and in 5 % CO_2_ (Gähwiler et al. 1997) and kept for 14 DIV (n = 12 for E12, n = 10 for E14, n = 17 for E18), 21 DIV (n = 11 for E12, n = 17 for E14, n = 19 for E18), 28 DIV (n = 11 for E12, n = 11 for E14, n = 8 for E18), and 35 DIV (n = 8 for E12, n = 11 for E14, n = 9 for E18). Fresh medium was supplied every 3–4 days.

### Culture medium

The sterilized culture medium was composed of 55 % DMEM, 32.5 % Hanks’ balanced salt solution (HBSS; Gibco, Stockholm, Sweden), 10 % fetal bovine serum (FCS; Gibco, Stockholm, Sweden), 1.5 % glucose (Gibco, Stockholm, Sweden) and 1 % Hepes (Gibco, Stockholm, Sweden). At plating, antibiotics (10,000 U/ml penicillin, 10 mg/ml streptomycin, 25 μg/ml amphotericin; Gibco, Stockholm, Sweden) were added to the medium to a final concentration of 1 % and from the first medium change antibiotics were excluded.

### Co-cultures of VM and GFP-positive cells

GFP-positive astrocytes obtained from the cortex of genetically modified postnatal day 2 (P2) Sprague–Dawley rat pups (β-actin-eGFP reporter line) were prepared as reported previously (East et al. 2009, 2013) and maintained in PDL-coated 75 cm^2^ flasks for 14 days, shaken for 4 h at 150 rpm to remove microglia and other less adherent cells before trypsinization and cryostorage in DMEM containing 10 % DMSO and 20 % FCS. After thawing, cells were mixed with medium containing 1 % antibiotics, plated on a pre-coated PDL Petri dish and incubated for 4 days at 37 °C. Then, to separate and discard all other cells from the astrocytes, attached to the bottom of the Petri dish, they were shaken at 300 rpm for 6 h. The remaining astrocytes were supplied with fresh medium and left at 37 °C for further proliferation. Medium was changed twice a week. At the time for initiation of the co-culture, the cells were trypsinized with 0.25 % trypsin–EDTA and centrifuged at 180×*g* for 5 min. Supernatant was aspirated and the cells were washed with fresh medium two times. Cells were thereafter re-suspended in the medium to final concentration of minimum 3.3 × 10^6^ cells/ml. 5 μl of the suspension and one VM tissue piece, dissected from E14 fetuses, were mixed in 20 μl of plasma together with 10 μl thrombin to form a clot. The rest of the procedure was performed as described above. These cultures were kept for 21 DIV.

### Immunohistochemistry

Single VM cultures were fixed at 14 DIV, 21 DIV, 28 DIV, and 35 DIV for 1 h in 2 % paraformaldehyde in 0.1 M phosphate buffered saline (PBS; pH = 7.4). The primary antibodies raised against tyrosine hydroxylase (TH; mouse anti-rat; diluted 1:1500; ImmunoStar, Hudson, WI, USA or rabbit anti-rat; diluted 1:300; Millipore AB, Solna, Sweden), vimentin (VIM; chicken anti-rat; diluted 1:800; Abcam, Cambridge, UK, or mouse anti-pig; diluted 1:200; Sigma-Aldrich, Stockholm, Sweden) were applied on cultures after rinsing them thoroughly in PBS, to visualize dopamine neurons and astrocytes, respectively. The use of two TH and two vimentin antibodies was due to technical reasons, both TH antibodies and both vimentin antibodies displayed however the same distribution pattern. The cultures were incubated with primary antibodies for 48–72 h in a humid chamber at 4 °C, followed by goat serum (5 %; Sigma-Aldrich, Stockholm, Sweden) as blocking solution for 15 min at room temperature, and subsequently with secondary antibodies, Alexa 594 (diluted 1:500; goat anti-mouse and goat anti-rabbit; Molecular Probes Inc., Eugene, OR, USA) and Alexa 488 (diluted 1:200; goat anti-chicken and goat anti-mouse; Molecular Probes Inc., Eugene, OR, USA), for 1 h at room temperature. For staining the cell nuclei, cultures were incubated in DAPI (diluted 1:50, Molecular Probes Inc., Eugene, OR, USA) for 10 min at room temperature. All antibodies and DAPI were diluted in 0.1 M PBS containing 1 % Triton X-100.

Similar protocol was used for staining of the VM-GFP-positive astrocytic co-cultures. Since the GFP-positive cells were green fluorescent, Alexa 355 (diluted 1:100; goat anti-mouse; Molecular Probes Inc., Eugene, OR, USA) was used as a secondary antibody against vimentin, for 1 h at room temperature. DAPI staining was not performed. All antibodies were diluted in 0.1 M PBS containing 1 % Triton X-100.

### Image analysis and statistical analysis

In each culture, astrocytic migration and TH-positive nerve fiber outgrowth from the periphery of the tissue slice to their distal end were measured using a scale mounted in one ocular of a fluorescence microscope. Astrocytic migration was measured in four perpendicularly placed directions and the distance to the outermost migrated astrocytes was determined. Four measurements were performed in areas with the longest nerve fiber outgrowth. Thereafter, the mean values, calculated for each culture, were used for statistical evaluation. Statistical analyses were performed using one-way ANOVA followed by Bonferroni post hoc test. The cultures were analyzed blind-coded. All results are expressed as means ± SEM. *p* < 0.05 was set as level of significance. Images were captured with a digital camera connected to a computer, and analyzed using Openlab software (Improvision; Coventry, UK).
